# Probing supramolecular structures in solution by resonant energy transfer in the X-ray range

**DOI:** 10.1039/d5sc05911a

**Published:** 2025-10-06

**Authors:** Viola C. D'mello, Venkateswara Rao Mundlapati, Jeremy Donon, Valérie Brenner, Michel Mons, Denis Céolin, Eric Gloaguen

**Affiliations:** a Laboratoire Interactions, Dynamiques et Lasers, Université Paris-Saclay, Commissariat à l'Énergie Atomique et Aux Énergies Alternatives, Centre National de la Recherche Scientifique CEA-Saclay 91191 Gif-sur-Yvette France; b Synchrotron SOLEIL, L'Orme des Merisiers BP 48, St Aubin 91192 Gif sur Yvette France denis.ceolin@synchrotron-soleil.fr; c Institut des Sciences Moléculaires d’Orsay, Université Paris-Saclay, Centre National de la Recherche Scientifique Rue André Rivière, Bât. 520 91400 Orsay France eric.gloaguen@cnrs.fr

## Abstract

Supramolecular structures in solution are probed using Far-Zone Resonant Energy Transfer (FZRET) in an aqueous potassium acetate microjet. This advanced X-ray spectroscopic technique relies on the resonant energy transfer between donor atoms, *i.e.* core-ionised potassium ions, and acceptor atoms a few nm away. These experiments reveal an inhomogeneous distribution of ions in water, and are consistent with the presence of nm-sized ionic clusters at 4.1 M concentration.

## Abstract 2

The first Far-Zone Resonant Energy Transfer (FZRET) spectroscopy experiment in liquid microjet provides quantitative characterisation of supramolecular organisation in solution with atomic spatial resolution.

## Graphical abstract

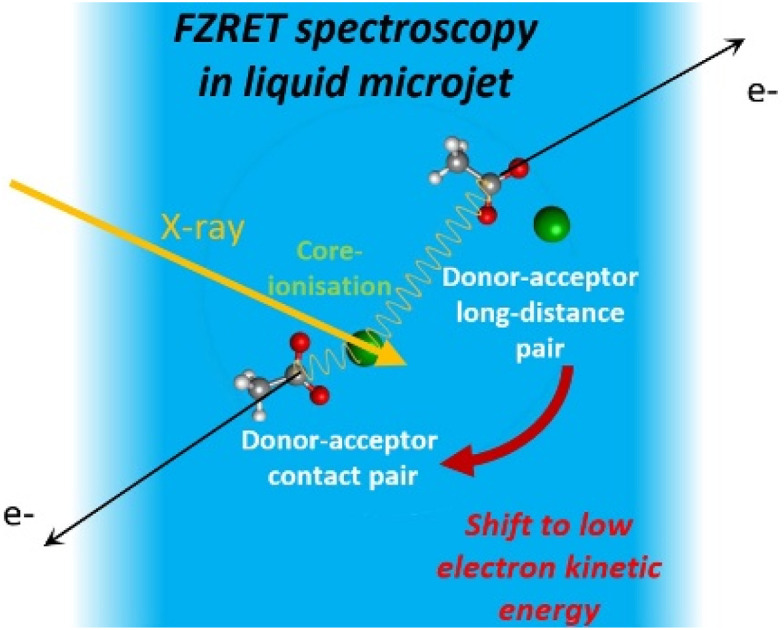

## Introduction

Supramolecules in solution are non-covalently bound species famously known for being at the heart of several processes like self-assembly or reactivity.^1,2^ They give rise to molecular selectivity and recognition,^3^ and their properties can ultimately be exploited to design molecular devices^4^ and/or mimic biological systems and functions.^5^ Their structural characterisation is often a challenge due to their intrinsic conformational complexity and dynamic nature, especially for those supramolecules that cannot be isolated and crystallised for analysis by X-ray diffraction. Structural information may then be obtained by combining *in situ* techniques^2,6^ like nuclear magnetic resonance (NMR), X-ray solution scattering, vibrational circular dichroism (VCD) and multidimensional spectroscopy, or *ex situ* gas phase techniques^7^ like ion mobility, mass spectrometry and IR spectroscopy.

In this context, liquid microjets provide a unique opportunity to probe the supramolecular structure *in situ* by using gas phase techniques.^8,9^ X-ray photons can penetrate these microjets and reach the bulk, where they may induce fluorescence^10^ or produce electrons of sufficiently high energy to escape into the gas phase with no or limited inelastic events,^11^ providing a direct probe of the ∼10 nm-thick outer layer of the microjet.^8,12^ Recent efforts on the measurement of the vertical ionisation energy (VIE) of liquid water illustrate the potential of this approach.^13^ Investigations of these solutions in microjets are mainly carried out by X-ray photoelectron spectroscopy (XPS), but the recent use of more advanced techniques, such as photoelectron circular dichroism (PECD),^14^ offer additional diagnostics.

**Fig. 1:**
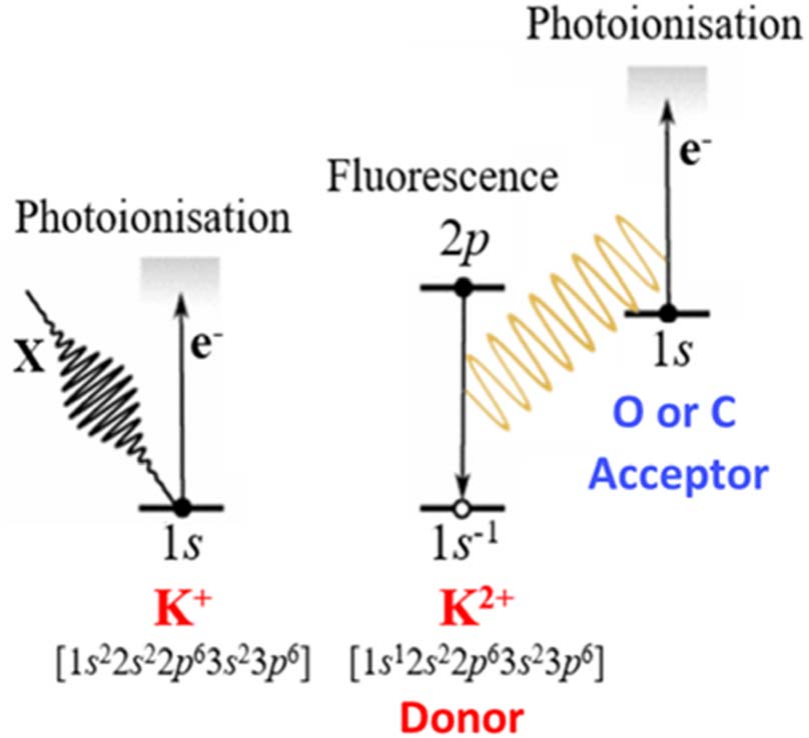
Sequence of events in (K^+^ → O or C) FZRET experiments.

Similarly, far-zone resonant energy transfer (FZRET) spectroscopy is a powerful technique, which has only been applied to gas and solid samples so far.^15^ The three-step mechanism of FZRET can be described as follows (Fig. 1): (i) an X-ray photon core-ionises a donor atom (D), (ii) the core hole in D^+^ can relax by X-ray fluorescence, (iii) which may ultimately core-ionise an acceptor atom (A) in a resonant energy transfer process. The Coulomb field generated by the hole of D^+^ modifies the ionisation energy of A according to the D–A distance, thereby shifting the emitted electron kinetic energy (EKE) to lower energies as compared to direct photoionisation.^15^ Applied to a liquid microjet, FZRET spectroscopy can potentially probe supramolecular structures in aqueous solutions with atomic spatial resolution.

In earlier reported FZRET measurements on gaseous and solid samples,^15^ two opposite behaviours were observed. These observations can be rationalised provided that the following considerations are taken into account: (1) the fraction of D–A pairs that contribute to the FZRET signal results from two antagonistic effects when the D–A distance increases, *i.e.* on one hand, the decrease of efficiency of the energy transfer and, on the other hand, the increasing number of D–A pairs; (2) among these pairs, those where the D^+^ hole is close enough to A to induce a significant and measurable Coulomb shift of its ionisation energies define a noteworthy subset designated as “short-distance” D–A pairs. These Coulomb shifts Δ*E* can be converted into D–A distances *d* using the Coulomb potential energy^16^ expressed in eqn (1).1
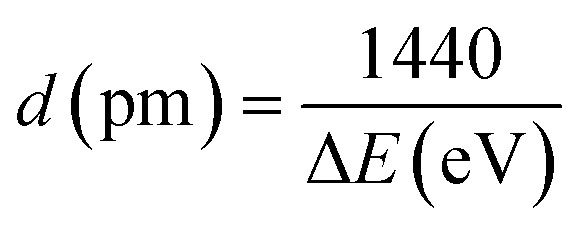


The interatomic distances thus obtained have an accuracy better than 20%.^15^ In solid CuO (D = Cu and A = O), the observed Cu → O FZRET signal was almost unshifted as compared to direct photoionisation (Δ*E* < 1 eV), pointing to a dominant contribution from “long-distance” D–A pairs.^15^ Indeed, in a solid sample where the density of donor and acceptor atoms is homogeneous, short-distance D–A pairs contributing to Coulomb shifts greater than 1 eV, *i.e.* those separated by less than ∼1.5 nm according to eqn (1), represent a negligible fraction of D–A pairs separated by only a few nm (*e.g.* 3% of the pairs separated by at most 5 nm). By contrast, an inhomogeneous distribution of the acceptors around donors may favour the contribution from short-distance relatively to long-distance D–A pairs. This was observed for gaseous SF_6_ where the S → F FZRET signal is dominated by intramolecular S–F pairs (SF bond length = 156 pm), and thus strongly shifted in energy by 10.9 eV.^15^ Therefore, one can anticipate that FZRET signals in solutions may display solid-like, gas-like or mixed behaviours, depending on the nature of the D–A pairs (solvent–solvent, solute–solvent, solvent–solute or solute–solute), and the solute concentration. At low concentration, solvent–solvent or solute–solvent pairs are expected to give an unshifted, solid-like FZRET signal. However, solute–solute D–A pairs, combined with factors introducing inhomogeneity, such as ion pairing, may result in shifted, gas-like FZRET signals. In general, FZRET spectroscopy is intrinsically suitable for investigating inhomogeneous media and their supramolecular organisation.

Therefore, the present study explores this uncharted territory of FZRET between two solutes in an aqueous solution, with the aim to document their self-assembly. Ion pairing being a pillar of supramolecular organisation,^17^ ionic reactions^18^ or biological function,^19^ electrolyte solutions are systems of choice for such investigations. Potassium acetate (KAcO) is particularly appropriate due to its high solubility in water, facilitating microjet experiments at high solute concentration. Also, KAcO solutions have been extensively studied both experimentally and theoretically. Considering K^+^ as the donor, the main experiments and their interpretation in terms of structure by quantum chemical calculations are presented as follows (full set of experiments available in the SI): K^+^ → O FZRET in a diluted KCl aqueous solution in order to characterise the fluorescence emitted by core-ionised potassium ions; XPS of O(1s) to characterise the microjet formed from a 3 M KAcO aqueous solution; K^+^ → O and K^+^ → C FZRET of the same solution to investigate K^+^–O and K^+^–C pairs, respectively.

## Methods

### Experimental

**Fig. 2:**
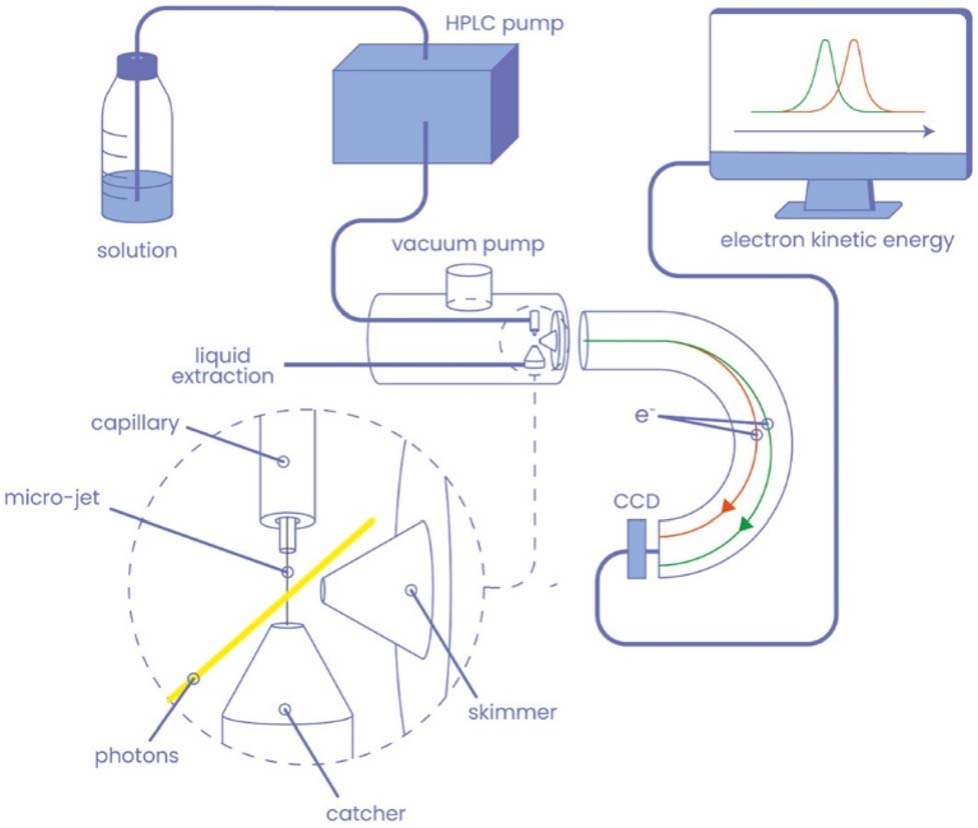
Liquid microjet configuration of the hard X-ray photoelectron spectroscopy (HAXPES) station of the GALAXIES beamline (SOLEIL synchrotron facility).

Experiments were performed using the microjet liquid environment (Fig. 2) installed on the HAXPES station of the GALAXIES beamline of the SOLEIL synchrotron facility.^20^ The head of the microjet is composed of a 30 μm-diameter glass capillary mounted vertically and facing a CuBe temperature-controlled catcher having a 300 μm-diameter opening. The catcher is placed at a distance of about 3 mm from the capillary and is permanently pumped in order to extract the liquid. The alignment of these elements is performed by piezo motors and visualised by a camera. The head of the microjet is inserted in a differential-pumped tube, itself connected to the spectrometer chamber with a 3-axes motorised manipulator. On this tube, two holes of 2 mm-diameter allow the photons to pass through, and a 500 μm-diameter hole skimmer allows the electrons created in the interaction region to be collected and directed towards the detector. The axis of the spectrometer lens is parallel to the horizontal polarisation axis of the light. The aqueous solutions are injected in the capillary using a HPLC pump with a flow of about 1 mL min^−1^. In these conditions, the pressure in the main chamber is kept below 10^−5^ mbar during the measurements. All solutions were prepared using deionised MilliQ water, and additional filtering and degassing procedures were performed. XPS and FZRET spectra are recorded by monitoring the electron signal as a function of their kinetic energy. The principle of (K^+^ → O or C) FZRET spectroscopy is shown on Fig. 1.

### Theoretical

The distance distributions of the different atom pairs considered in this work are extracted from previously reported calculations.^17^ The theoretical approach used was fully described in previous publications, and is available in the SI Materials.^17,21^ Briefly, the potential energy surface of (AcO^−^, K^+^)·(H_2_O)_527_ clusters whose density was set at 1.02 (*i.e.* that of a 1 M potassium acetate solution at room temperature and atmospheric pressure)^22^ by freezing the outer layers, were explored using the AMOEBA polarisable force field^23^ coupled with a global minima search method based on a biased Monte–Carlo algorithm developed by Scheraga.^24^ Simulations were parametrised in order to ensure a sufficient exchange between contact ion pairs (CIPs) and solvent-shared ion pairs (SIPs). Although not originally targeted, ions separated by 2 solvation shells (2SIPs) were also found. In the end, distance distributions were built from the lowest-energy structures among the 150 × 10^3^ generated, *i.e.* 29 × 10^3^ CIPs, 5 × 10^3^ SIPs and 1.5 × 10^3^ 2SIPs.

## Results and discussion

### K^2+^(1s^−1^) Kα_1,2_ fluorescence characterisation

In the experiments reported in this article, potassium ions are FZRET donors. Hence, we first need to characterise the Kα_1,2_ fluorescence following core-ionisation of K^+^. To that end, a microjet produced from a KCl 1 M aqueous solution was investigated by FZRET spectroscopy. In practice, photon energies above the (1s) ionisation threshold of K^+^ at 3611.9 eV lead to (1s)-hole formation by photoionisation.^25^ The resulting K^2+^(1s^−1^) species may follow a 2p → 1s relaxation through ^2^S → ^2^P_3/2_ (Kα_1_) or ^2^S → ^2^P_1/2_ (Kα_2_) transitions, resulting in the Kα fluorescence doublet. The acceptors are oxygen atoms of water (A = O_w_), that are homogeneously distributed around the K^+^ donors. Therefore, this K^+^ → O FZRET spectrum is expected to be dominated by the contribution of long-distance D–A pairs as in CuO (*vide supra*), leading to an unshifted FZRET signal made of a doublet as a result of the ionisation of O_w_(1s) by the Kα_1,2_ fluorescence. Indeed, the spectrum recorded by shining 3800 eV photons on the KCl solution microjet (Fig. S3.2) consists of such a doublet at EKEs 2774.0 and 2777.0 eV with a 2 : 1 intensity ratio in accordance with the respective multiplicities of the ^2^P_3/2_ and ^2^P_1/2_ states of K^2+^(2p^−1^). Knowing the VIE of O_w_(1s) (538.1 eV),^26^ we can deduce the Kα_1_ and Kα_2_ of K^2+^(1s^−1^) to be at 3315.1 eV and 3312.1 eV respectively. These values are slightly shifted to higher energies relative to those measured for solid potassium, *i.e.* 3313.95 eV and 3311.19 eV.^27^ These differences primarily reflect the variations in energy levels between K^2+^ and K^+^.

### Characterisation of the microjet produced from a KAcO 3 M aqueous solution by XPS

**Fig. 3:**
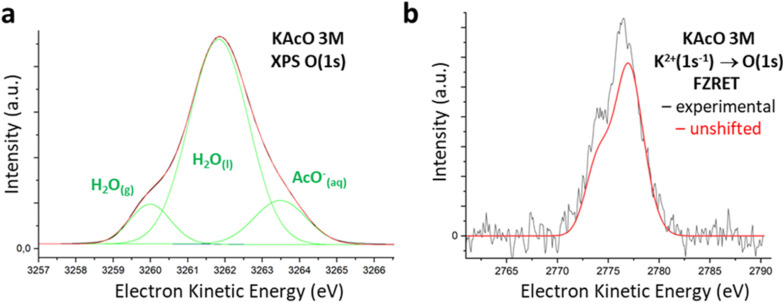
Spectra recorded on a microjet produced from a KAcO 3 M aqueous solution. (a) XPS spectrum in the O(1s) spectral region at photon energy 3799.9 eV (black). Experimental data were fitted by the sum (red) of three Gaussian functions (green) which are assigned to the three types of O atoms, H_2_O_(g)_, H_2_O_(l)_ and AcO^−^_(aq)_. Both experimental (black) and fitted (red) spectra are almost identical. (b) Baseline subtracted K → O FZRET spectrum (black) at photon energy 3800 eV. The contribution of long-distance D–A pairs to the FZRET signal (unshifted FZRET) is shown in red (see text and SI for explanations about signal construction and scaling).

The microjet generated from a 3 M KAcO aqueous solution was first investigated by XPS at a photon energy of 3800 eV in the O(1s) EKE range (Fig. 3a). The signal could be fitted (S2) by three contributions corresponding to three different chemical environments around the O atoms in this experiment. The components at 3260.0 and 3261.8 eV were assigned to H_2_O_(g)_ and H_2_O_(l)_ by comparison with the XPS spectrum of KCl (S2). The third band at 3263.5 eV was assigned to AcO^−^_(aq)_. The gaseous water molecules are formed due to microjet evaporation as the liquid phase is indeed out-of-equilibrium under vacuum conditions. Interestingly, the observed intensity ratio between AcO^−^_(aq)_ and H_2_O_(l)_ corresponds to an apparent average concentration of 4.1 M (S2), which is significantly larger than the initial concentration (3 M). As suggested by XPS spectra (Fig. 3a and S2), the substantial water evaporation may explain, by itself, an accumulation of non-volatile solutes near the surface, leading to a radial concentration gradient and a concentration increase in the ∼10 nm thick outer layer of the microjet probed by XPS. Therefore, 4.1 M is taken as the effective average concentration of KAcO in the solution probed.

### Probing O(1s^−1^) by FZRET

**Fig. 4:**
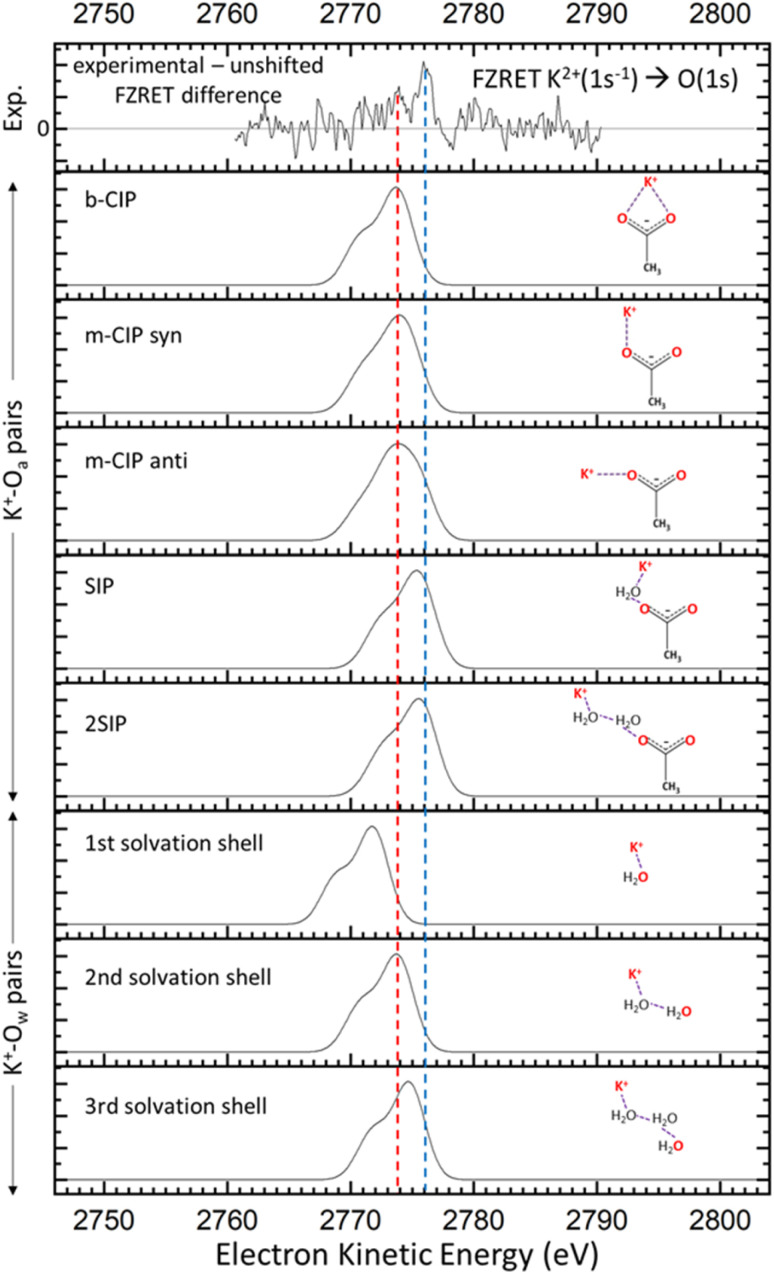
The difference between experimental and reconstructed unshifted K → O FZRET spectra (top panel) is compared to the predicted FZRET signals of K^+^–O pairs (atoms in red) in several supramolecular structures: K^+^–O_a_ pairs in bidentate contact ion pairs (b-CIP), monodentate contact ion pairs in a *syn*/*anti* geometry respectively (*m*-CIP *syn*/*anti*), solvent-shared ion pairs (SIP), and ion pairs separated by two solvation shells (2SIP) (intermediate panels); K^+^–O_w_ pairs for the first three solvation shells of K^+^ (lower panels). Coloured dashed lines help the comparison between experimental and theoretical data.

The next step was to record K^+^ → O FZRET spectra in the KAcO microjet (Fig. 3b, S3.1 and S3.4). As in the KCl solution (Fig. S3.2), we expect the observation of FZRET between K^+^–O_w_ pairs, with a contribution from water vapour remaining negligible due to its relatively low density. We also expect an additional signal coming from K^+^–O_a_ pairs, where O_a_ are the oxygen atoms of acetate anions. For these solute–solvent and solute–solute D–A pairs, two factors favour the observation of shifted FZRET signals at sufficiently high concentration: (i) ion pairing occurs (*e.g.* CIPs are formed above 2 M concentration),^17^ increasing the energy-shifted contribution of short-distance K^+^–O_a_ pairs *versus* long-distance pairs; (ii) high ion concentrations tend to decrease the number of K^+^–O_w_ pairs, (∼11 water molecules for each cation at 4.1 M, instead of ∼58 in a KCl 1 M solution),^28^ while ion solvation always favours short-distance K^+^–O_w_ pairs. However, it is impossible to know *a priori* if the population of these short-distance ion pairs will be significant enough to detect an energy-shifted component in the FZRET signal. Therefore, it is critical to know the FZRET signal with no Coulomb shift and compare it to the observed spectrum to deduce the energy-shifted contribution. This unshifted FZRET signal corresponding to the contribution of long-distance K^+^–O pairs can be reconstructed from the K^2+^(1s^−1^) Kα_1,2_ fluorescence as characterised by FZRET in KCl, and the vertical ionisation energies of O_w_(1s) and O_a_(1s) obtained from the XPS experiment (S3.3). The structure of this unshifted FZRET signal (red curve in Fig. 3b) essentially resembles the K^2+^(1s^−1^) Kα_1,2_ fluorescence doublet, the difference between the VIE of O_w_(1s) and O_a_(1s) being significantly smaller than the doublet splitting. It becomes clear from Fig. 3b that the experimental spectrum contains an additional contribution on the low-energy flank where one expects the signal from short-distance K^+^–O pairs. This contribution consists mainly of two peaks at 2776.0 eV and 2773.9 eV as shown in S3.4 and top panel of Fig. 4.

To assign this difference signal to short-distance K^+^–O pairs, one needs to consider the corresponding distance distributions in solution. We have previously carried out conformational explorations of ion pair supramolecular structures using the polarisable force field AMOEBA combined with a non-local optimisation method.^17^ This work provides the theoretical K^+^–O distance distributions expected for each ion pair type in solution (S3.5). We converted these distance distributions into EKE distribution using eqn (1), and further convoluted with the Kα_1,2_ fluorescence doublet to obtain the FZRET signal expected for each K^+^–O pair type (Fig. 4). Remarkably, the EKE range corresponds to that of the short-distance K^+^–O pairs. The peak at 2773.9 eV Fig. 4 (red dashed line) may be assigned either to the three types of K^+^–O_a_ CIPs, or K^+^–O_w_ pairs of the second solvation shell of K^+^. The former assignment is more likely as (i) CIPs must be present at this concentration,^17^ and (ii) the peak shape in the 2770–2775 eV EKE range matches that calculated for the CIPs. Moreover, the latter assignment can be dismissed for two reasons: the average number of water molecules per cation and anion (*i.e.* ∼11)^17^ is barely enough to complete the first solvation shell of both ions (6 for K^+^,^29^ and 5 or 6 for only the carboxylate group of acetate^30^), (ii) we see no signal ascribable to K^+^–O_w_ pairs of the first solvation shell, making the consideration of the second solvation shell irrelevant. This analysis suggests that K^+^ ions are mostly in contact with O_a_ atoms rather than O_w_ at this ∼4.1 M concentration. The peak at 2776.0 eV (blue dashed line in Fig. 4) lies at a slightly higher EKE than that expected for FZRET between the most distant K^+^–O pairs considered in the simulations, *i.e.* 0.6 nm for K^+^–O_a_ (Fig. S3.5.2), and 0.7 nm for K^+^–O_w_ (Fig. S3.5.3). Therefore, this signal corresponds to short-distance K^+^–O pairs producing a measurable energy-shifted FZRET signal, but not short enough to document the local structure around potassium ions. Given the small shift of this peak relative to the unshifted FZRET signal (<1 eV), it may then be assigned to K^+^–O pairs separated by at least 1.5 nm. Remarkably, there is no peak at lower EKE values that can be assigned to K^+^–O_w_ pairs, the shortest distance detected for these pairs therefore being ∼1.5 nm.

### Probing C(1s^−1^) by XPS and FZRET

**Fig. 5:**
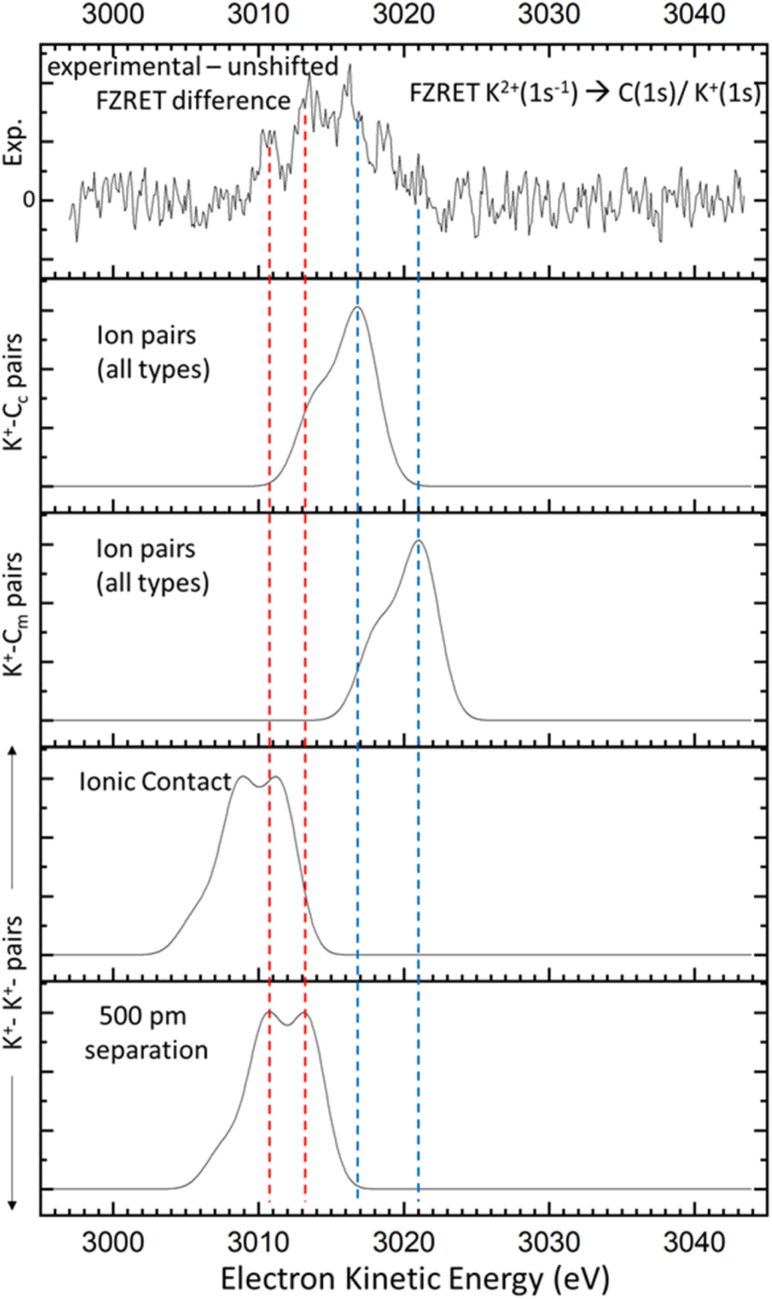
The difference between experimental and reconstructed unshifted K^+^ → K^+^/C FZRET spectra (top panel) is compared to the predicted FZRET signals in several supramolecular structures: K^+^–C_c_ and K^+^–C_m_ pairs in all types of ion pairs (CIPs, SIPs and 2SIPs, intermediate panels); K^+^–K^+^ pairs in case of ionic contact and a 500 pm separation (lower panels). Coloured dashed lines help the comparison between experimental and theoretical data.

The C(1s) spectral region was also investigated by XPS (Fig. S4). The spectrum shows a quadruplet peak, revealing four ionising processes. The C(1s) ionisation of acetate carboxylate (C_c_) and methyl (C_m_) carbon atoms gives the peaks at 3506.3 and 3509.8 eV. Ionisation of K^+^(2p) appears as a doublet at 3498.9 and 3501.7 eV corresponding to the ^2^P_1/2_ and ^2^P_3/2_ states respectively. From this data, we extract the respective VIEs to build the unshifted K^+^ → K^+^/C FZRET spectrum, following the same methodology as for the K^+^ → O case (S5). This unshifted FZRET signal is compared with the observed FZRET spectrum (Fig. S5.2.2). The difference spectrum (Fig. 5, top panel) displays a rather broad and structured signal, with a low EKE feature at ∼3011 eV. The expected FZRET signal between K^+^–C_c_ and K^+^–C_m_ pairs was extracted using the same theoretical dataset as for K^+^–O pairs. These signals lie in the high-energy side of the difference FZRET signal (blue dashed lines in Fig. 5), which is consistent with the presence of ion pairs in solution, as revealed by the K^+^ → O FZRET measurements. Considering the shortest separation, and thus the smallest EKE of K^+^–C_c_/C_m_ pairs, we deduce that these pairs cannot be assigned to the low-energy side of the difference FZRET signal. However, K^+^–K^+^ pairs may potentially contribute to this energy range. This is supported by the calculated FZRET spectrum for two potassium ions separated by 300 pm (*i.e.* twice the ionic radius 151 pm).^31^ Remarkably, the theoretical signal for K^+^–K^+^ separated by 500 pm matches the low-energy side of the difference FZRET signal, including the feature at 3011 eV (red dashed lines in Fig. 5). These potassium ions are quite close to each other, but apparently not as close as in potassium acetate crystals (∼400 pm).^32^ However, this result is at the current limit of the approach, since the Coulomb shifts estimated by eqn (1) gave an error of ∼20% in the S–F distance in gaseous SF_6_ obtained using FZRET. This point highlights the need for a more robust model to capture more quantitative structural details of the supramolecular structures in solutions using FZRET spectroscopy.

## Conclusions

In conclusion, FZRET measurements enabled us to detect the following species around K^+^ ions in the KAcO aqueous solution: O_a_ in contact with K^+^, other K^+^ lying at ∼0.5 nm, and finally O_w_ at a distance of at least ∼1.5 nm from K^+^. While contacts between K^+^ and O_a_ are expected at 4.1 M concentration, the absence of signal from O_w_ within the ∼1.5 nm sphere around K^+^, and the simultaneous detection of other K^+^ ions in this sphere, point to an inhomogeneous distribution of these three constituents in solution. Indeed, these results are consistent with the presence of nm-size ionic clusters of potassium acetate separated by water molecules. FZRET measurements on a microjet therefore have the demonstrable ability to characterise supramolecular arrangements in solutions at an unprecedented atomic spatial resolution.

## Author contributions

V. C. D., V. R. M., J. D., M. M., D. C. and E. G. performed experimental investigations. V. B., M. M., D. C., and E. G. contributed to the formal analysis. D. C. and E. G. acquired the funding and conceptualized the project. D. C. and E. G. wrote the original draft. D. C. was in charge of the experimental data curation and resources. E. G. supervised the project.

## Conflicts of interest

There are no conflicts to declare.

## Supplementary Material

SC-OLF-D5SC05911A-s001

## Data Availability

The data supporting this article have been included as part of the supplementary information (SI). Supplementary information: full set of experiments, procedures, and data analyses. See DOI: https://doi.org/10.1039/d5sc05911a.
