# Supplementary material for: Probing supramolecular structures in solution by resonant energy transfer in the X-ray range
Source: Chem Sci. 2025 Oct 6;16(44):21041–6. doi: 10.1039/d5sc05911a (PMC12517049; doi:10.1039/d5sc05911a)
Supplement: SC-016-D5SC05911A-s001 [file SC-016-D5SC05911A-s001.pdf]

Supplementary Information (SI) for Chemical Science.
This journal is © The Royal Society of Chemistry 2025

Supplementary Information

Probing Supramolecular Structures in Solution by Resonant Energy Transfer in the X-ray range

Viola C. D'mello, Venkateswara Rao Mundlapati, Jeremy Donon, Valérie Brenner, Michel Mons, Denis Céolin, Eric Gloaguen

This Supplementary Information provides details about experiments, procedures, and analyses needed for a proper interpretation of the main FZRET experiments. It includes:

- Electron Kinetic Energy (EKE) calibration (S1)
- photon energy calibration (S2)
- measurement of the average concentration of the part of the microjet actually probed
- extraction of the FZRET signals (S3.1 for the O(1s) spectral region, S5.2 for the K^+(2p)/C(1s) spectral region)
- measurement of the $K\alpha_{1,2}$ fluorescence of $K^{2+}(1s^{-1})$ (S3.2)
- construction of the unshifted FZRET reference signals (S3.3 for the O(1s) spectral region ; S5.1 for the K^+(2p)/C(1s) spectral region)
- extraction of the shifted FZRET signal component (S3.4 for the O(1s) spectral region; S5.2 for the K^+(2p)/C(1s) spectral region)
- theoretical distance distributions for FZRET signal assignment (S3.5 and S5.3)

S1 KLL Auger spectra of K^+ - EKE Calibration

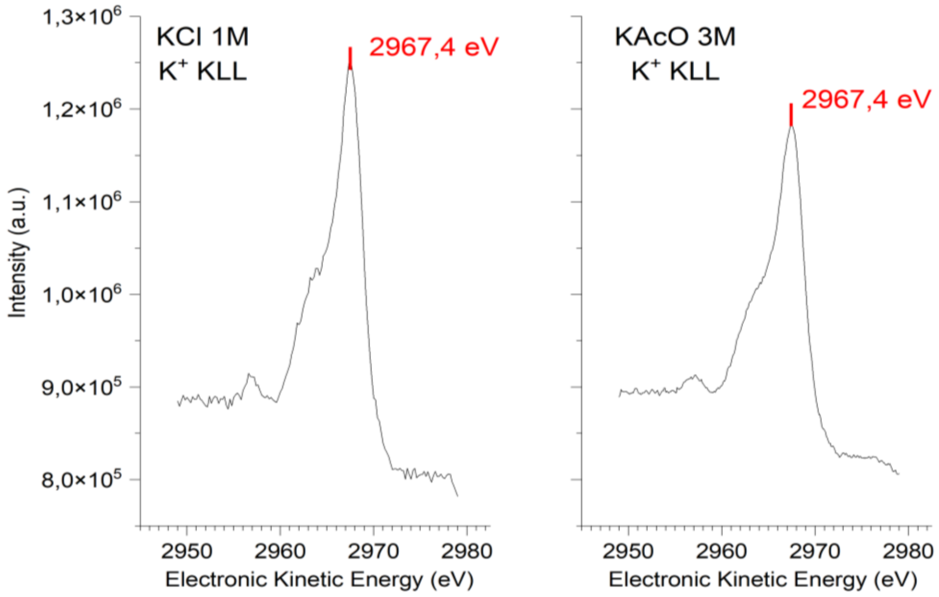

Figure S1: KLL Auger spectra of K^+ in KCl 1 M (left) and KAcO 3 M (right) solutions after EKE calibration. Photon energy is set at 3800 eV in both cases.

KLL Auger electrons from K^+, *i.e.* electrons resulting from relaxation of the photoinduced $K^+(1s^{-1})$ hole towards two 2p core vacancies, are recorded for KCl 1 M and KAcO 3 M solutions (Figure S1) after excitation by 3800 eV photons. These electrons are found to peak at 2967.4 eV,[1] which has been used to calibrate the EKE axes for both spectra.

Interestingly, the calibration applied is the same in both experiments (-1.0 eV). According to recent measurements of electron extraction work functions inherent to the interface,[2] it has been shown that these ones are responsible for a 0.5 eV increase of VIEs of O(1b1) relative to neat water upon adding 25 mM of a surfactant (tetrabutyl ammonium), whereas no measurable change occurs for a 2 M sodium iodide aqueous solution. The same study demonstrated that this 0.5 eV change of the extraction work function results specifically from the accumulation of the surfactant at the surface of the microjet. Given that there is no measurable difference between K^+ KLL Auger EKE from KCl 1 M and KAcO 3 M solutions, the surfaces of these microjets behave like that of neat water within our measurement uncertainty. Other said, there is no measurable EKE difference indicating that ion accumulation at the surface of the KAcO 3 M microjet leads to an organized layer inducing a surfactant effect, which can be further neglected.

S2 XPS spectra in the O(1s) spectral region – Photon energy calibration and average concentration measurement

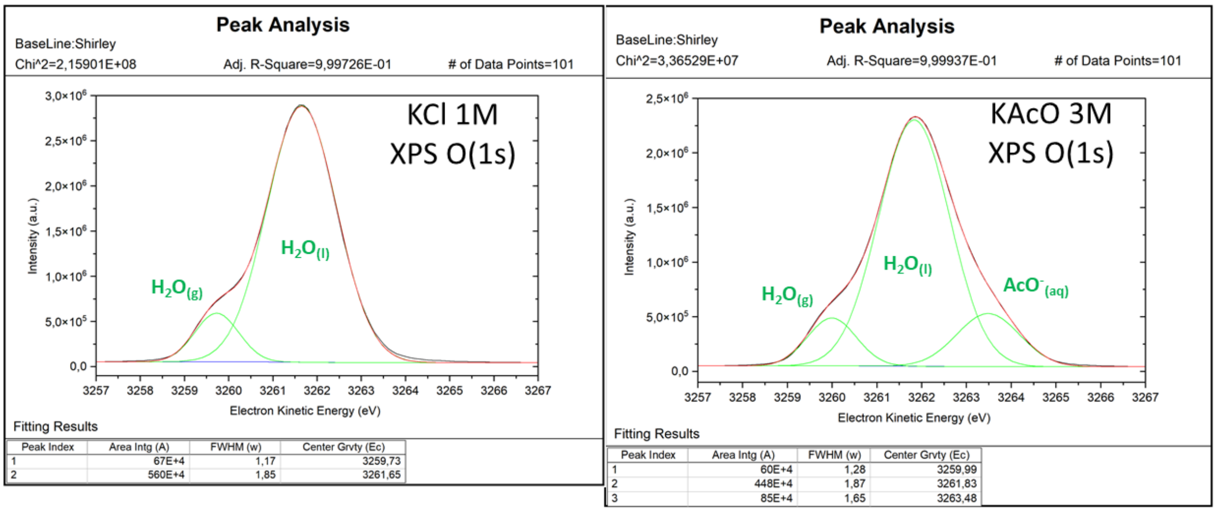

Figure S2: XPS spectra in the O(1s) spectral region (black) for microjets produced from KCl 1 M (left) and KAcO 3 M (right) solutions after EKE calibration (*cf.* section 2.1). Spectra are recorded for photon energies set at 3800 eV (respectively 3799.7 and 3799.9 eV after photon calibration). After Shirley's non-linear baseline subtraction (baseline shown in blue),[3] experimental data are fitted by sums (red) of Gaussian functions (green) whose parameters are shown in the tables below each spectra. Both experimental (black) and fitted (red) spectra are almost identical. See text for explanations.

Spectra are fitted by sums of Gaussian functions $G(E)$ defined as:

$$G(E) = \frac{Ae^{-\frac{4\ln(2)(E-E_C)^2}{w^2}}}{w\sqrt{\frac{\pi}{4\ln(2)}}}$$

where *Ec* is the energy of the band center, *w* the full width at half maximum (FWHM), and *A* the total area.

For the KCl 1 M solution XPS spectrum $S_{XPS\ O(1s)\ \cdot\ KCl\ 1M\ \cdot\ 3800}$ (Fig S2 left), two types of photoelectron are expected: those resulting from (1s) ionisation of oxygen atoms from water molecules (O_w) in the liquid microjet, noted $H_2O_{(l)}$, and those resulting from the ionisation of water molecules in the gas surrounding the microjet, noted $H_2O_{(g)}$, the latter having less EKE due to a relatively larger stabilisation of the ion state than the ground state in the liquid phase compared to the gas phase.[4] The spectrum is then fitted by the sum of two Gaussian functions:

$$S_{XPS\ O(1s)\cdot KCl\ 1M\cdot 3800} = G^{H_2O_{(g)}}_{XPS\ O(1s)\cdot KCl\ 1M\cdot 3800} + G^{H_2O_{(l)}}_{XPS\ O(1s)\cdot KCl\ 1M\cdot 3800}$$

Parameters resulting from the fit are displayed at the bottom of Fig S2. In general, the number of significant digits of these values depends on the signal-to-noise ratio of the spectrum. In practice, this number is determined from the error provided by the fitting procedure.

Given the vertical ionisation energy (VIE) of $O_w(1s)$ electrons in solution (538.1 eV)[5], one determines the calibrated photon energies from the measurement of the EKE of $H_2O_{(l)}$ (Fig. S2) as follows:

$$E^{light}_{XPS\ O(1s)\cdot KCl\ 1M\cdot 3800} = E_c^{H_2O_{(l)}} + 538.1 = 3799.7\ eV$$

For the KAcO 3 M solution XPS spectrum $S_{XPS\ O(1s)\cdot KAcO\ 3M\cdot 3800}$ (Fig S2 right), a third contribution is expected, detected as a high energy shoulder, and assigned to photoelectrons coming from the (1s) ionisation of oxygen atoms (O_a) from aqueous acetate, $AcO^-_{(aq)}$. Therefore, three Gaussian functions are used to fit the spectrum:

$$S_{XPS\ O(1s)\cdot KAcO\ 3M\cdot 3800} = G^{H_2O_{(g)}}_{XPS\ O(1s)\cdot KAcO\ 3M\cdot 3800} + G^{H_2O_{(l)}}_{XPS\ O(1s)\cdot KAcO\ 3M\cdot 3800} + G^{AcO^-_{(aq)}}_{XPS\ O(1s)\cdot KAcO\ 3M\cdot 3800}$$

This minimum number of Gaussian functions is sufficient to fit almost perfectly the experimental signal, enabling us to determine accurately all the nine fitting parameters. The calibrated photon energy can be determined as above:

$$E^{light}_{XPS\ O(1s)\cdot KAcO\ 3M\cdot 3800} = E_c^{H_2O_{(l)}} + 538.1 = 3799.9\ eV$$

In turn, the VIE of $O_a(1s)$ electrons is deduced from the measurement of EKE of $AcO^-_{(aq)}$ (Fig S2 right) : 3799.9 - 3263.5 = 536.4 eV.

Furthermore, these fits are useful to determine the spectral width of the $O_w(1s)$ transition in this experiment, which is further needed in the data analysis.

$$w^{H_2O_{(l)}}_{XPS\ O(1s)\cdot KCl\ 1M\cdot 3800} \cong w^{H_2O_{(l)}}_{XPS\ O(1s)\cdot KAcO\ 3M\cdot 3800} \cong 1.86\ \pm 0.01\ eV$$

Finally, the water:acetate intensity ratio is $A^{H_2O_{(l)}}_{XPS\ O(1s)\ \cdot KAcO\ 3M} : A^{AcO^-_{(aq)}}_{XPS\ O(1s)\ \cdot KAcO\ 3M}$ = 5.3. However, the relative proportion of these two types of oxygen atom, $r_{Ow/Oa}$, can be calculated for a 3 M solution as follows:

$$n_{water}M_{water} + n_{AcOK}M_{AcOK} = \frac{dn_{AcOK}}{C_{AcOK}}$$

$$r_{O_w/O_a} = \frac{n_{water}}{2n_{AcOK}} = \frac{\frac{d}{C_{AcOK}} - M_{AcOK}}{2M_{water}}$$

where n_i is the amount of compound i, M_i its molecular mass, C_i its concentration, and d is the density of the solution. From the density of a 3 M potassium acetate solution, *i.e.* 1132 g L^{-1} (extrapolated from ref.[6]), one finds that $r_{Ow/Oa}$ = 7.8. The band intensity ratio being significantly smaller, *i.e.* 5.3, and the ionisation efficiency between both atom types being expected similar, one must conclude that the measured signal corresponds to an apparently more concentrated solution than 3 M. A higher concentration is a direct consequence of water evaporation of the microjet, which is evidenced by the

$H_2O_{(g)}$ signal. If one includes this $H_2O_{(g)}$ signal when estimating the intensity ratio, one finds $(A^{H_2O(l)}_{XPS\ O(1s)\ \cdot KAcO\ 3M} + A^{H_2O(g)}_{XPS\ O(1s)\ \cdot KAcO\ 3M}) : A^{AcO^-_{(aq)}}_{XPS\ O(1s)\ \cdot KAcO\ 3M}$ = 5.9, which is still below the expected value of 7.8. One likely cause is that most of the evaporated water molecules are not in the region illuminated by the synchrotron light. Another reason could be the uneven detection of photoelectrons emitted at or near the surface relatively to that generated deeper in the microjet due to a weaker transmission in the condensed phase. In presence of a positive concentration gradient from the core to the surface of the microjet, the observed $r_{Ow/Oa}$ would indeed be smaller than expected. Such a concentration gradient is likely given water evaporation from the surface of the microjet. In any case, the value $r_{Ow/Oa}$ = 5.3 enables us to estimate the average concentration of 4.1 M as a result of water evaporation. It should be noted that this latter value is only a rough estimate based on the density of a solution at room temperature and standard pressure,[6] which may be different from that of the microjet.

S3 FZRET photoelectron spectra in the O(1s) spectral region

S3.1 FZRET signal assignment and extraction

The FZRET signal resulting from the O (1s) ionisation by the $K\alpha_{1,2}$ fluorescence line emitted by $K^{2+}(1s^{-1})$ is recorded to characterise this fluorescence for the same solutions as above (Fig. S3.1). Assignment of these signals is confirmed by two experiments:

- Changing the photon energy above the $K^+(1s)$ ionisation threshold (measured at 3611.9 eV)[1] from 3800 to 4000 eV does not shift the EKE of the observed signal.
- Changing the photon energy below the $K^+(1s)$ ionisation threshold at 3400 eV supresses the signal.

Both FZRET signals overlap with another much broader and more intense background signal increasing with the EKE. In addition, the relative intensities on either side of the FZRET signal, *i.e.* a positive offset of the electron signal to the high EKE side, is opposite to what is expected from electron scattering.[3] Without any evidence of the process responsible for this effect, and given the superimposition of several signals, one arbitrarily chooses to apply a baseline correction relying on purely mathematical grounds. In practice, one defines a non-linear background subtraction by taking into account both the change of slope and offset of the apparently linear baselines below and above the FZRET signal, which is delimited by two arbitrary energies $\mathbf{E_A}$ and $\mathbf{E_B}$ respectively (Figure S3.1). The total baseline used for baseline subtraction is thus made of two linear parts, one below $\mathbf{E_A}$ and one above $\mathbf{E_B}$, and a polynomial connection part between $\mathbf{E_A}$ and $\mathbf{E_B}$ based on the unique third order polynomial connecting **A** and **B** with the same slope as the linear parts in **A** and **B**.

This baseline subtraction procedure is applied similarly for all the FZRET spectra presented further.

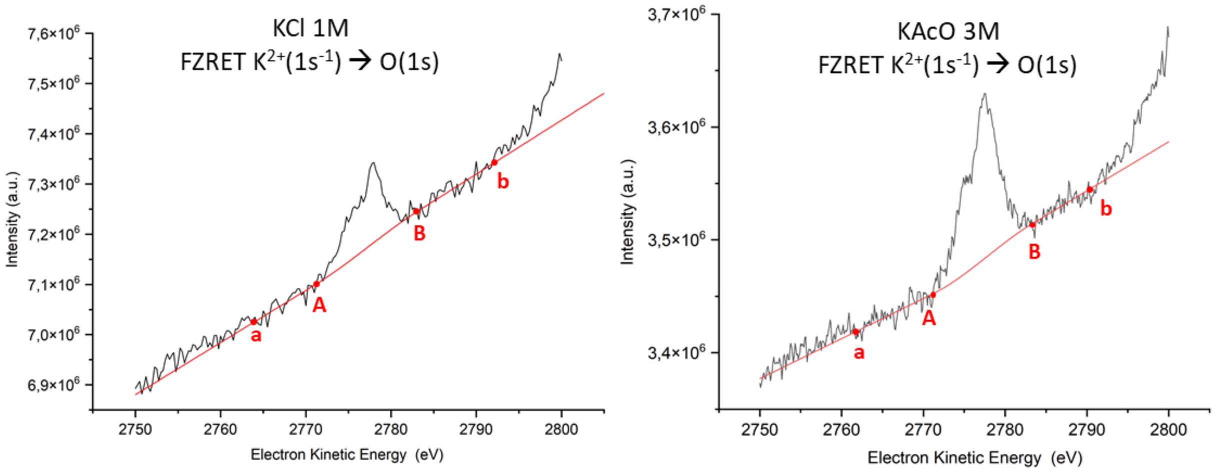

Figure S3.1: Raw photoelectron spectra (black) recorded in the spectral region expected for a FZRET signal corresponding to the O(1s) ionisation by the $K\alpha_{1,2}$ photons emitted by the $K^{2+}(1s^{-1})$ ions produced by a 3800 eV synchrotron irradiation of a microjet formed from KCl 1 M (left) and KAcO 3 M (right) solutions. The total baseline (red) is composed of two linear parts, *i.e.* below point **A** (fitted between points **a** and **A**) and above point **B** (fitted between points **b** and **B**), and a polynomial part (between **A** and **B**), see text for explanations. EKE axes are not calibrated.

S3.2 KCl FZRET signal analysis - $K^{2+}(1s^{-1})$ $K\alpha_{1,2}$ fluorescence characterisation

The $K\alpha_{1,2}$ synchrotron light induced X-ray fluorescence emitted by $K^{2+}(1s^{-1})$ corresponds to the K(2p) → (1s) relaxation of one electron. This relaxation involves two transitions $^2S \rightarrow {}^2P_{1/2}$ and $^2S \rightarrow {}^2P_{3/2}$ responsible for the fluorescence doublet. For such a KCl 1 M solution, donor atoms (D = K^+) are within a condensed phase where acceptors (A = O_w) are rather homogeneously distributed around donor atoms. Therefore, the FZRET spectrum $S_{FZRET\ O(1s)\cdot KCl\ 1M}$ resulting from the ionisation of O(1s) water molecules (Figure S3.2) is dominated by the contribution of long-distance donor-acceptor (D-A) pairs, as observed previously for solid CuO (D = Cu, A = O).[7]

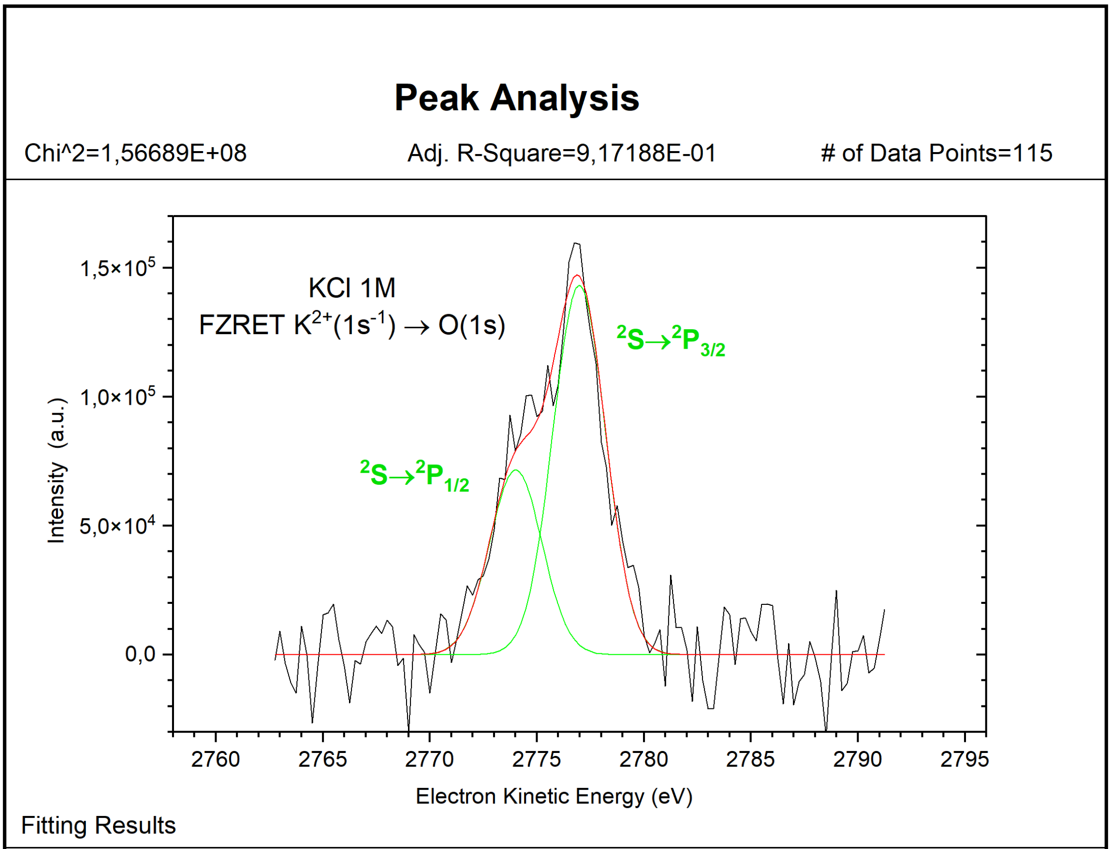

Peak Index	Area Intg (A)	FWHM (w)	Center Grvty (Ec)	Area IntgP (%)
1	22E+4	2,9	2 774,0	33
2	44E+4	2,9	2 777,0	67

Figure S3.2: Baseline subtracted FZRET $K^{2+}(1s^{-1}) \rightarrow O(1s)$ spectrum of a microjet produced from a KCl 1 M solution (black) after EKE calibration. Photon energy is set at 3800 eV. Experimental data are fitted by sums (red) of Gaussian functions (green), see text for explanations.

Consequently, a doublet made of two Gaussian functions is used to fit the signal as follows:

$$S_{FZRET\ O(1s)\cdot KCl\ 1M} = G_{FZRET\ O(1s)\cdot KCl\ 1M}^{^2S\rightarrow{}^2P_{1/2}\cdot H_2O_{(l)}} + G_{FZRET\ O(1s)\cdot KCl\ 1M}^{^2S\rightarrow{}^2P_{3/2}\cdot H_2O_{(l)}}$$

The multiplicity of the electronic term ^2P leads us to impose a constraint on the area of the Gaussian functions, *i.e.* $A_{FZRET\ O(1s)\cdot KCl\ 1M}^{^2S\rightarrow{}^2P_{3/2}\cdot H_2O_{(l)}} = 2A_{FZRET\ O(1s)\cdot KCl\ 1M}^{^2S\rightarrow{}^2P_{1/2}\cdot H_2O_{(l)}}$. In addition, the widths result from the inhomogeneous broadening of these two atomic lines, and are therefore assumed to be identical: $w_{FZRET\ O(1s)\cdot KCl\ 1M}^{^2S\rightarrow{}^2P_{1/2}\cdot H_2O_{(l)}} \cong w_{FZRET\ O(1s)\cdot KCl\ 1M}^{^2S\rightarrow{}^2P_{3/2}\cdot H_2O_{(l)}}$. Consequently, only four independent variables are determined from the fit.

The determination of $Ec^{^2\mathrm{S}\rightarrow{}^2\mathrm{P}_{3/2}\cdot H_2O_{(l)}}_{FZRET\ O(1s)\cdot KCl\ 1M}$ and $Ec^{^2\mathrm{S}\rightarrow{}^2\mathrm{P}_{1/2}\cdot H_2O_{(l)}}_{FZRET\ O(1s)\cdot KCl\ 1M}$ together with the knowledge of the VIE of O(1s) electrons of water molecules (538.1 eV)[5] enables us to estimate the photon energies of the $K\alpha_{1,2}$ fluorescence of $K^{2+}(1s^{-1})$, *i.e.* 2774.0 + 538.1 = 3312.1 ± 0.2 and 2777.0 + 538.1 = 3315.1 ± 0.1 eV for the $^2S \rightarrow {}^2P_{1/2}$ and $^2S \rightarrow {}^2P_{3/2}$ transitions respectively.

The width of each component $w^{^2\mathrm{S}\rightarrow{}^2\mathrm{P}\cdot H_2O_{(l)}}_{FZRET\ O(1s)\cdot KCl\ 1M}$ measured at 2.9 eV is significantly larger than the width obtained in the XPS experiments (1.86 eV, *cf.* above), which demonstrates that each component of this fluorescence source is spectrally broader than the synchrotron source.

Considering that among all sources of spectral broadening, only the light differs between XPS O(1s) and FZRET O(1s) experiments, one obtains the following relationship between the FWHM of the light source at 3800 eV, $w_{light\cdot 3800} \cong 0.45$ eV, and the FWHM of each component of the $K\alpha_{1,2}$ fluorescence doublet of $K^{2+}(1s^{-1})$, $w_{light\cdot {}^2\mathrm{S}\rightarrow{}^2\mathrm{P}\cdot KCl\ 1M}$:

$$w_{light\cdot {}^2\mathrm{S}\rightarrow{}^2\mathrm{P}\cdot KCl\ 1M} = \sqrt{\left[w^{^2\mathrm{S}\rightarrow{}^2\mathrm{P}\cdot H_2O_{(l)}}_{FZRET\ O(1s)\cdot KCl\ 1M}\right]^2 - \left(\left[w^{H_2O_{(l)}}_{XPS\ O(1s)\cdot KCl\ 1M\cdot 3800}\right]^2 - \left[w_{light\cdot 3800}\right]^2\right)}$$

one can estimate that:

$$w_{light\cdot {}^2\mathrm{S}\rightarrow{}^2\mathrm{P}\cdot \mathrm{KCl\ 1M}} \cong 2.3\ eV$$

S3.3 Simulated unshifted FZRET spectrum in KAcO

For interpretation purposes, one needs to know the FZRET spectrum when acceptor atoms O(1s) are ionised far enough from the donor $K^{2+}(1s^{-1})$, *i.e.* when the EKE of the emitted electrons from the acceptor is not modified by the electric field of the donor. It is possible to build such a reference spectrum, *i.e.* the unshifted FZRET spectrum corresponding the contribution of long-distance D-A pairs to the total FZRET spectrum, by combining results from the previous measurements as demonstrated below.

The XPS spectrum of KAcO 3 M solution, $S_{XPS\ O(1s)\cdot KAcO\ 3M\cdot 3800}$ (Section S2), shows that two types of electrons are emitted from the solution. However, in a FZRET experiment, the light source is the $K\alpha_{1,2}$ fluorescence doublet of $K^{2+}(1s^{-1})$, thus doubling the number of Gaussian functions contributing to the total spectrum. This reference spectrum $S_{FZRET\ O(1s)\ \cdot KAcO\ unshifted}$ is then composed of four Gaussian functions:

$$S_{FZRET\ O(1s)\cdot KAcO\ unshifted} = G^{^2\mathrm{S}\rightarrow{}^2\mathrm{P}_{1/2}\ \cdot H_2O_{(l)}}_{FZRET\ O(1s)\cdot KAcO\ unshifted} + G^{^2\mathrm{S}\rightarrow{}^2\mathrm{P}_{1/2}\cdot AcO^-_{(aq)}}_{FZRET\ O(1s)\cdot KAcO\ unshifted} +$$

$$G^{^2\mathrm{S}\rightarrow{}^2\mathrm{P}_{3/2}\ \cdot H_2O_{(l)}}_{FZRET\ O(1s)\ \cdot KAcO\ unshifted} + G^{^2\mathrm{S}\rightarrow{}^2\mathrm{P}_{3/2}\cdot AcO^-_{(aq)}}_{FZRET\ O(1s)\ \cdot KAcO\ unshifted}$$

Each Gaussian function being defined by three parameters, *A*, *Ec* and *w*, this reference spectrum is thus defined by 4*3=12 variables.

First, let us consider the four parameters governing the areas. The relative area between the $H_2O_{(l)}$ and $AcO^-_{(aq)}$ components result from the ion concentration as measured by the XPS spectrum $S_{XPS\ O(1s)\cdot KAcO\ 3M\cdot 3800}$, *i.e.* 5.3 (S2). In addition, the relative intensities of both components of the

fluorescence doublet (1:2 ratio) is fixed. Overall, all the relative intensities of the four Gaussian functions are known, leaving only one free parameter corresponding to the total signal intensity.

Regarding the centres of the Gaussian functions, they result directly from the knowledge of the corresponding two EKE that are extracted from (i) the KCl FZRET experiment (S3.2), *i.e.* 2774.0 and 2777.0 eV for the O_w(1s) electrons, and (ii) the XPS experiment presented in S2 giving the relative position of O_a(1s) 1.6 eV higher than O_w(1s), leading to 2775.6 and 2778.6 eV.

Finally, the widths of the Gaussian functions are expected to be the same as that observed in the KCl FZRET experiment $S_{FZRET\ O(1s)\cdot KCl\ 1M}$, *i.e.* 2.9 eV (S3.2), as both the X-fluorescence light source $K^{2+}(1s^{-1})$, the ionisation process considered, and the detector are identical.

In the end, 11 of the 12 parameters needed to build the spectrum $S_{FZRET\ O(1s)\ \cdot KAcO\ unshifted}$ are known from the experiments presented above. The last parameter controls the total intensity of this reference spectrum, which is presented on Fig. S3.3 together with the details of its different contributions. This spectrum appears like an unresolved doublet, although it is actually a quadruplet.

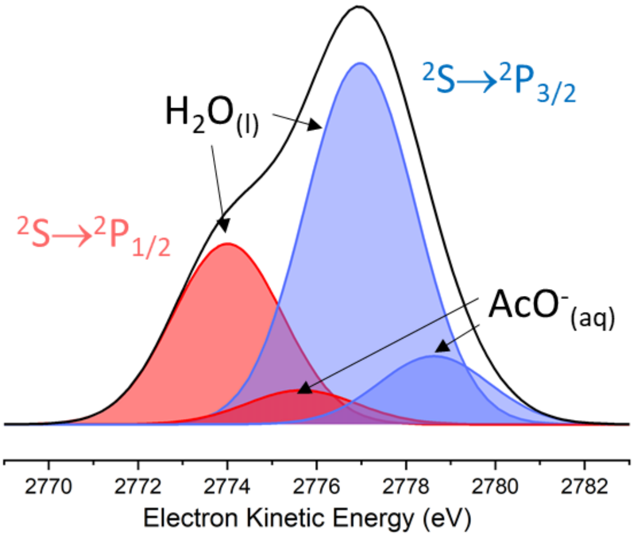

Figure S3.3: Simulated FZRET $K^{2+}(1s^{-1}) \rightarrow$ O(1s) spectrum of the long-distance D-A pairs in a 4.1 M KAcO solution (black). The contributions of electrons resulting from the ionisation from each component of the $K\alpha_{1,2}$ fluorescence doublet of $K^{2+}(1s^{-1})$ are shown in red ($^2S \rightarrow {}^2P_{1/2}$) and blue ($^2S \rightarrow {}^2P_{3/2}$). Each contribution is made of a doublet due to the two O(1s) types of atoms in solution, as observed in figure S2 right for XPS, with, however, a slightly worse resolution due to a spectrally broader X-fluorescence than the synchrotron source (*cf.* S2).

S3.4 KAcO FZRET signal decomposition

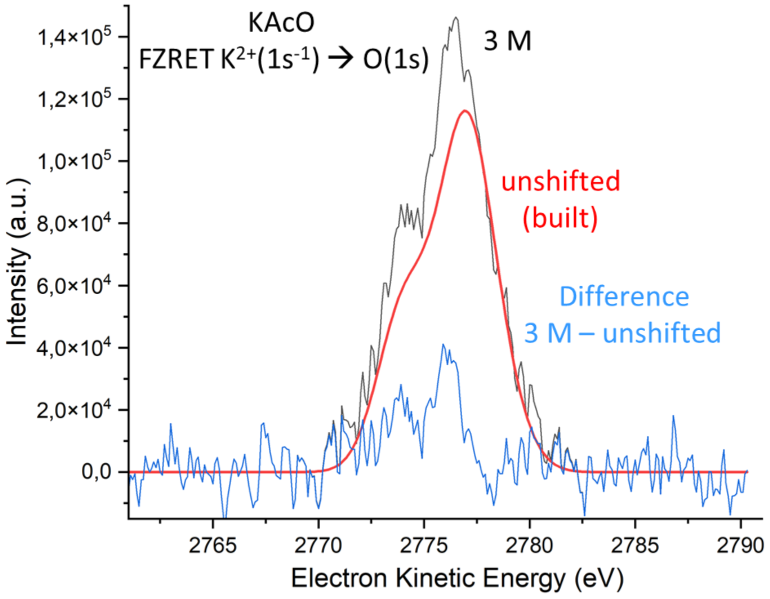

Figure S3.4 Baseline subtracted FZRET $K^{2+}(1s^{-1}) \rightarrow O(1s)$ spectrum of a KAcO 3 M solution (black). Photon energy is set at 3800 eV. This spectrum can be decomposed in two contributions, that of the long-distance D-A pairs known from the unshifted FZRET spectrum (red, *cf.* S3.3), and the remaining difference, which corresponds to short-distance D-A pairs (blue). See text for explanations.

The baseline subtracted FZRET spectrum of a KAcO 3 M solution $S_{FZRET\ O(1s)\cdot KAcO\ 3M}$ is presented on Fig. S3.4 together with the unshifted FZRET spectrum $S_{FZRET\ O(1s)\ \cdot KAcO\ unshifted}$ already presented in Fig. S3.3. $S_{FZRET\ O(1s)\cdot KAcO\ 3M}$ contains the additional, shifted contribution of short-distance D-A pairs compared to $S_{FZRET\ O(1s)\ \cdot KAcO\ unshifted}$. In order to extract the signal of short distance D-A pairs, $S_{FZRET\ O(1s)\cdot KAcO\ 3M-unshifted}$, one adjusts the relative intensity of $S_{FZRET\ O(1s)\cdot KAcO\ 3M}$ and $S_{FZRET\ O(1s)\ \cdot KAcO\ unshifted}$ in order to maximise their overlap, while keeping the difference signal always positive (Figure S3.4). From this procedure, one extracts the minimal contribution of the shifted signal to the total FZRET signal, avoiding the risk of overestimation of the shifted part. Remarkably, such a procedure leads to a good agreement between the unshifted and total signals on the high EKE side, where the unshifted signal is indeed the only expected contribution in this EKE range. This good agreement suggests that the shifted contribution presented here is not significantly underestimated.

S3.5 K^+-O pairs theoretical distance distributions for assignment of the KAcO FZRET signal in the O(1s) region

Specific D-A (*i.e.* K^+-O) distances are expected in KAcO solutions, where one may distinguish between K^+-O_w and K^+-O_a distances. According to simulations previously carried out for (K^+, AcO^-) ion pairs in large water clusters (see Theoretical Methods),[8] the following theoretical distance distributions D_d are available: $D_d(K^+\text{-}O_w)$ for the first solvation shells; $D_d(K^+\text{-}O_a)$ for each type of ion pairing (Figures S3.5.1, S3.5.2 and S3.5.3).

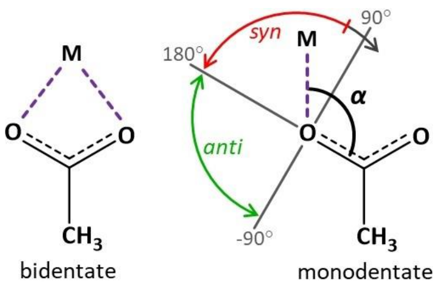

Figure S3.5.1 Schemes representing the bidentate (left) and monodentate (right) syn and anti contact ion pairs between an alkali cation (M) and acetate (reproduced from Ref[8])

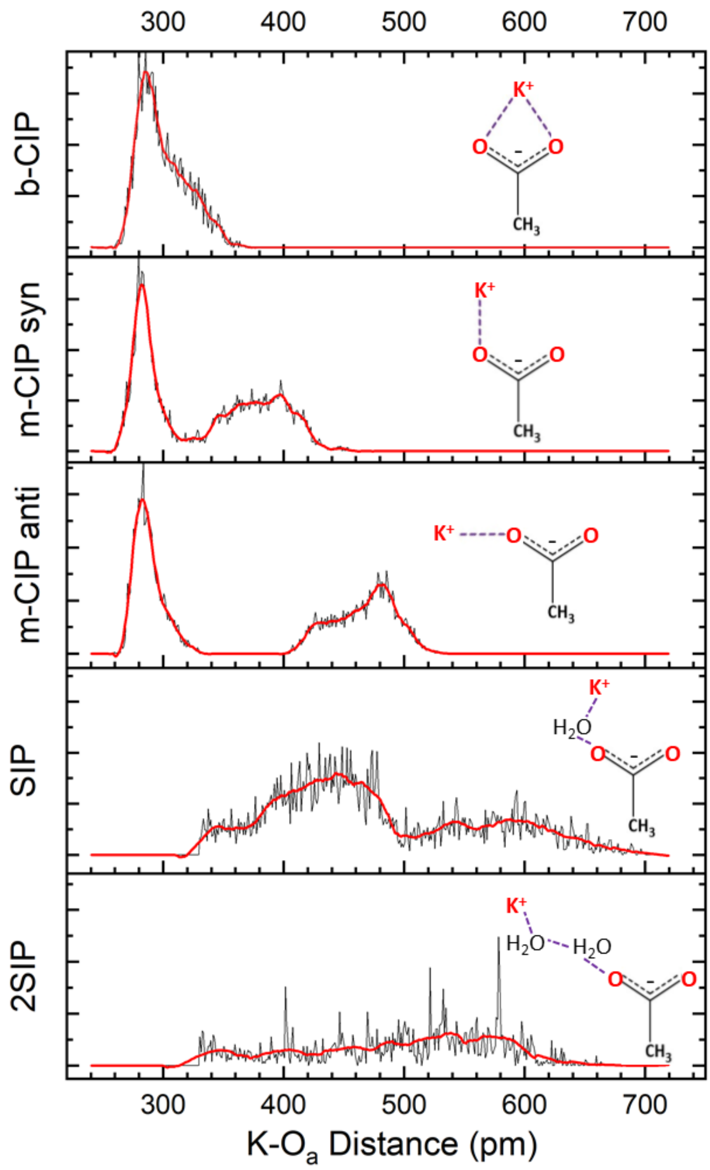

Figure S3.5.2 K^+-O_a distance distributions between atoms in red for *e.g.* bi- or mono-dentate, syn and anti contact ion pairs (CIPs), solvent-shared ion pairs (SIPs), and ions separated by 2 solvation shells (2SIPs), taken from ref[8]. Raw distributions are in black. Noise resulting from limited sampling is reduced by using smoothed distributions (red).

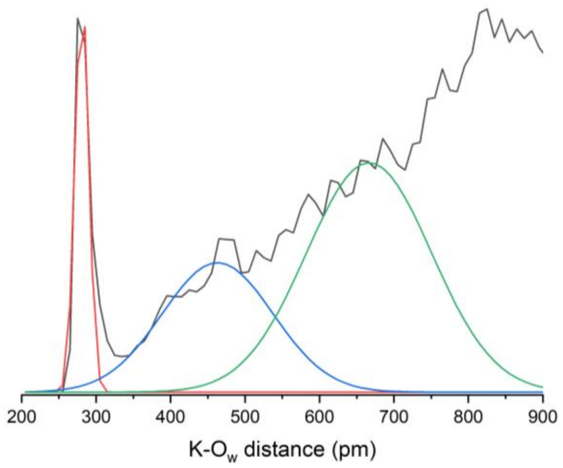

Figure S3.5.3 K^+-O_w distance distribution (black) decomposed into the first (red), second (blue) and third (green) solvation shells, taken from ref[8].

Assuming that the EKE shift relative to the corresponding unshifted FZRET transitions, ΔE, results from a simple Coulomb shift, the interatomic distance (d) may then converted into EKE shift, following the equation:

$$d(pm) = \frac{1440}{\Delta E(eV)}$$

Consequently, all distance distributions, D_d, can be turned into energy distributions, D_E. Taking into account the doublet nature of the X-ray fluorescence and its spectral width, one obtains a specific theoretical FZRET signal for each distance distribution presented above. Eventually, this theoretical set of FZRET signals can be compared with the difference between 3 M and unshifted FZRET signals as shown in Figure 3.

S4 XPS spectrum in the K$^+$(2p)/C(1s) spectral region.

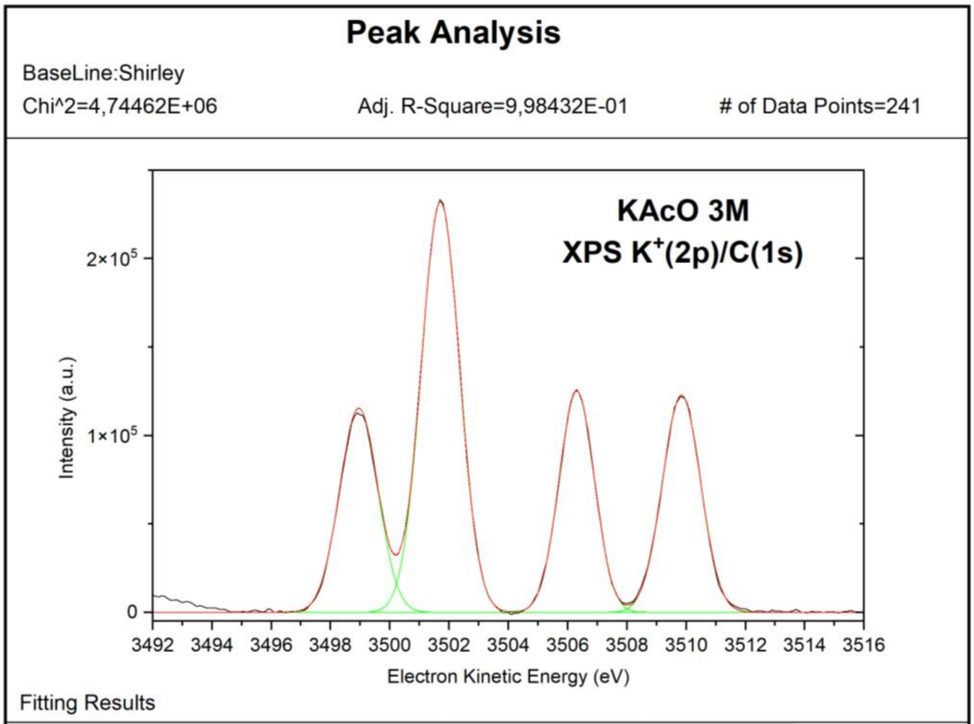

Fitting Results

Peak Index	Area Intg (A)	FWHM (w)	Center Grvty (Ec)	Area IntgP (%)
1	186E+3	1,52	3498,95	19,8
2	372E+3	1,51	3501,71	39,6
3	186E+3	1,39	3506,31	19,7
4	197E+3	1,51	3509,84	20,9

Figure S4: XPS spectrum in the K$^+$(2p)/C(1s) spectral region (black) of a microjet produced from a KAcO 3 M solution, recorded at a photon energy set at 3800 eV after Shirley's non-linear baseline subtraction[3] and EKE calibration. Experimental data are fitted by sums (red) of Gaussian functions (green) whose parameters are shown in the tables below the spectrum. See text for explanations.

The C(1s) ionisation of acetate produces two types of electrons corresponding to the respective ionisation of carbon atoms from carboxylate, C_c, or methyl groups, C_m. In addition, the VIEs of K$^+$(2p) are expected to be close to that of C(1s) according to the following reference values: 284.2 eV for C(1s);[9] 297.74 and 300.55 eV for aqueous K$^+$(2p).[10] Given the respective weight of the K$^+$(2p) states and their EKE expected near 3800 - 300 = 3500 eV, the doublet observed at 3498.95 and 3501.71 eV is assigned to their ionisation. Therefore, the XPS spectrum in this spectral region, $S_{XPS\,K^+(2p)/C(1s)\,\cdot\,KAcO\,3M\,\cdot\,3800}$ (Fig S4), is fitted by the sum of four Gaussian functions:

$$S_{XPS\,K^+(2p)/C(1s)\;\cdot\,KAcO\,3M\,\cdot\,3800} = G_{XPS\,K^+(2p)\;\cdot\,KAcO\,3M\,\cdot\,3800}^{\mathrm{K}^+(2p)^2\mathrm{P}_{1/2}} + G_{XPS\,K^+(2p)\;\cdot\,KAcO\,3M\,\cdot\,3800}^{\mathrm{K}^+(2p)^2\mathrm{P}_{3/2}} +$$

$$G_{XPS\,C(1s)\;\cdot\,KAcO\,3M\,\cdot\,3800}^{CO_2^-} + G_{XPS\,C(1s)\;\cdot\,KAcO\,3M\,\cdot\,3800}^{CH_3}$$

As a consequence of the assignment of the K$^+$(2p) doublet, the fit of the spectrum of Fig. S4 is carried out with a constraint, which forced the area of the second Gaussian function to be twice that of the first one in order to reflect the relative weights of the K$^+$(2p) states.

Given the higher polarity of the CO bond compared to CH, the VIE of C(1s) electrons is expected to be higher for C_c than for C_m. Therefore, the band at 3506.31 eV (resp. 3509.84 eV) is thus assigned to the ionisation of C_c(1s) (resp. C_m(1s)). In the end, the VIEs of C_m(1s), C_c(1s), K$^+$(2p) $^2P_{3/2}$ and K$^+$(2p) $^2P_{1/2}$ are

respectively measured at 290.16, 293.69, 298.29 and 301.05 eV. These later VIEs measured for aqueous K^+(2p) are larger by 0.5 eV than those reported previously, where energy calibration has been done using the VIE of the valence X-state of liquid water as a reference.[10] However, this latter VIE has long been a matter of controversy with different measurements spread over ~0.5 eV,[2] and may explain the difference with our measurement. We also cannot rule out an absolute error due to the lack of calibration of the photon energy in this experiment.

Knowing the VIE of K^+(1s) measured at 3611.9 eV,[1] the VIE difference between the final states $K^{2+}(1s^{-1})$ and $K^{2+}(2p^{-1})\ ^2P_{1/2}$ is thus 3611.9 - 301.0 = 3310.9 eV (resp. 3611.9 - 298.3 = 3313.6 eV between $K^{2+}(1s^{-1})$ and $K^{2+}(2p^{-1})\ ^2P_{3/2}$). These values are close, but different to that of the $K^{2+}(1s^{-1}) \rightarrow K^{2+}(2p^{-1})$ fluorescence doublet (i.e. 3312.1 and 3315.1 eV). Indeed, in the former case, VIE's provide orbital energy levels of the K^+ ion, whereas, in the latter case, fluorescence occurs between the relaxed orbitals of the K^{2+} ion.

In addition, these fits enable us to determine the spectral width of the transitions, which are further needed in the analysis:

$$w_{XPS\ K^+(2p)\ \cdot KAcO\ 3M\ \cdot 3800}^{\mathrm{K}^+(2p)^2\mathrm{P}_{1/2}} = w_{XPS\ K^+(2p)\cdot KAcO\ 3M\ \cdot 3800}^{\mathrm{K}^+(2p)^2\mathrm{P}_{3/2}} = 1.51\ \pm 0.01\ eV$$

$$w_{XPS\ C(1s)\ \cdot KAcO\ 3M\ \cdot 3800}^{CO_2^-} = 1.39\ \pm 0.01\ eV$$

$$w_{XPS\ C(1s)\ \cdot KAcO\ 3M\ \cdot 3800}^{CH_3} = 1.51\ \pm 0.01\ eV$$

S5 FZRET photoelectron spectra in the K^+(2p)/C(1s) spectral region

S5.1 Simulated unshifted FZRET spectrum

A simulated unshifted FZRET reference spectrum, $S_{FZRET\ K^+(2p)/C(1s)\ \cdot KAcO\ unshifted}$, can be defined and built in the K^+(2p)/C(1s) spectral region by following the same steps as for the O(1s) region (S3.3). In a FZRET experiment, the light source is the $K\alpha_{1,2}$ fluorescence doublet of $K^{2+}(1s^{-1})$, thus doubling the number of Gaussian functions contributing to the total spectrum as compared to the XPS experiment where a quadruplet is observed (S4). The FZRET reference spectrum $S_{FZRET\ K^+(2p)/C(1s)\ \cdot KAcO\ unshifted}$ is then composed of eight Gaussian functions:

$$S_{FZRET\ K^+(2p)/C(1s)\ \cdot KAcO\ unshifted} =$$

$$G_{FZRET\ K^+(2p)\cdot KAcO\ unshifted}^{^2\mathrm{S}\rightarrow ^2\mathrm{P}_{1/2}\cdot K^+(2p)^2P_{1/2}} + G_{FZRET\ K^+(2p)\cdot KAcO\ unshifted}^{^2\mathrm{S}\rightarrow ^2\mathrm{P}_{1/2}\cdot K^+(2p)^2P_{3/2}} + G_{FZRET\ C(1s)\cdot KAcO\ unshifted}^{^2\mathrm{S}\rightarrow ^2\mathrm{P}_{1/2}\cdot CO_2^-} + G_{FZRET\ C(1s)\cdot KAcO\ unshifted}^{^2\mathrm{S}\rightarrow ^2\mathrm{P}_{1/2}\cdot CH_3} +$$

$$G_{FZRET\ K^+(2p)\cdot KAcO\ unshifted}^{^2\mathrm{S}\rightarrow ^2\mathrm{P}_{3/2}\cdot K^+(2p)^2P_{1/2}} + G_{FZRET\ K^+(2p)\cdot KAcO\ unshifted}^{^2\mathrm{S}\rightarrow ^2\mathrm{P}_{3/2}\cdot K^+(2p)^2P_{3/2}} + G_{FZRET\ C(1s)\ \cdot KAcO\ unshifted}^{^2\mathrm{S}\rightarrow ^2\mathrm{P}_{3/2}\cdot CO_2^-} + G_{FZRET\ C(1s)\ \cdot KAcO\ unshifted}^{^2\mathrm{S}\rightarrow ^2\mathrm{P}_{3/2}\cdot CH_3}$$

Each Gaussian function being defined by three parameters, *A*, *Ec* and *w*, this reference spectrum is thus defined by 8*3=24 variables.

First, let us consider the eight parameters governing the areas. The fluorescence doublet (photon energies of 3312.1 and 3315.1 eV, S3.2) and the photon energy (3311.4 eV) used to record the K^+(2p)/C(1s) XPS spectrum (3800 eV) are both well larger than the VIEs considered (S4). It is then legitimate to consider that the relative intensities of the quadruplet measured in S4 are conserved in

the reference spectrum for both components of the fluorescence doublet. In addition, the relative intensities of both components of the fluorescence doublet (1:2 ratio) is fixed. Overall, all the relative intensities of the eight Gaussian functions are known, leaving only one free parameter corresponding to the total signal intensity.

Regarding the eight centres of the Gaussian functions, they result directly from the knowledge of the four VIEs extracted from the XPS experiment presented in S4, and of the photon energies of the fluorescence doublet (S3.2).

Finally, the widths of the Gaussian functions can be determined as follows, taking the example of the $C_m(1s)$ ionisation by the $^2S \rightarrow {}^2P_{1/2}$ component of the fluorescence doublet. First, several effects contribute to the total width: the spectral width of the light source w_{light}, the intrinsic width of the ionisation process considered w_{int}, and the width due to the apparatus w_{app} used for the measurement. The Gaussian function resulting from the convolution of these effects, one has the following relationship:

$$w_{FZRET\ C(1s)\ \cdot KAcO\ unshifted}^{{}^2S \rightarrow {}^2P_{1/2} \cdot CH_3} = \sqrt{w_{light \cdot {}^2S \rightarrow {}^2P \cdot KAcO}^2 + w_{int\ \cdot C(1s)\ CH_3}^2 + w_{app}^2}$$

Similarly, three widths measured in the previous experiments can be decomposed as follows:

$$w_{XPS\ C(1s)\ \cdot KAcO\ 3M \cdot 3800}^{CH_3} = \sqrt{w_{light \cdot 3800}^2 + w_{int\ \cdot C(1s)\ CH_3}^2 + w_{app}^2}$$

$$w_{FZRET\ O(1s) \cdot KAcO\ unshifed}^{{}^2S \rightarrow {}^2P \cdot H_2O_{(l)}} = \sqrt{w_{light \cdot {}^2S \rightarrow {}^2P\ \cdot KAcO}^2 + w_{int\ \cdot O(1s)\ H_2O_{(l)}}^2 + w_{app}^2}$$

$$w_{XPS\ O(1s) \cdot KAcO\ 3M \cdot 3800}^{H_2O_{(l)}} = \sqrt{w_{light \cdot 3800}^2 + w_{int\ \cdot O(1s)\ H_2O_{(l)}}^2 + w_{app}^2}$$

For the same reasons as in S3.3, $w_{FZRET\ O(1s) \cdot KAcO\ unshifed}^{{}^2S \rightarrow {}^2P \cdot H_2O_{(l)}}$ is equivalent to $w_{FZRET\ O(1s) \cdot KCl\ 1M}^{{}^2S \rightarrow {}^2P \cdot H_2O_{(l)}}$. Then, one finds the following relationship, which enables us to determine one of the eight widths needed to build $S_{FZRET\ K^+(2p)/C(1s)\ \cdot KAcO\ unshifted}$:

$$w_{FZRET\ C(1s) \cdot KAcO\ unshifted}^{{}^2S \rightarrow {}^2P_{1/2} \cdot CH_3} =$$

$$\sqrt{\left[w_{XPS\ C(1s)\ \cdot KAcO\ 3M \cdot 3800}^{CH_3}\right]^2 + \left[w_{FZRET\ O(1s) \cdot KCl\ 1M}^{{}^2S \rightarrow {}^2P \cdot H_2O_{(l)}}\right]^2 - \left[w_{XPS\ O(1s) \cdot KAcO\ 3M \cdot 3800}^{H_2O_{(l)}}\right]^2}$$

Similarly, all the other widths can be calculated with analogous formula.

In the end, 23 of the 24 parameters needed to build the spectrum $S_{FZRET\ K^+(2p)/C(1s)\ \cdot KAcO\ unshifed}$ are known from the experiments presented above. The last parameter controls the total intensity of the spectrum, which is presented on Fig. S5.1 together with the details of its different contributions. This spectrum appears like an unresolved quintuplet, although it is actually an octuplet.

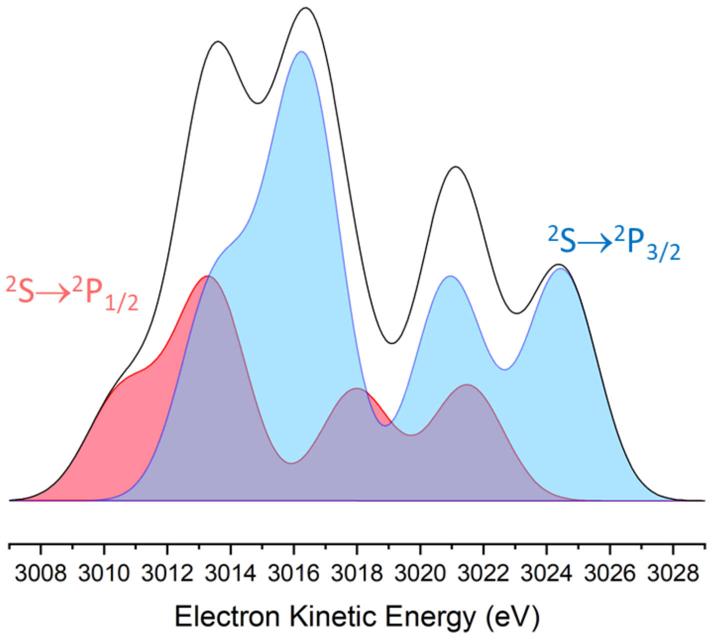

Figure S5.1: Simulated FZRET $K^{2+}(1s^{-1}) \rightarrow K^{+}(2p)/C(1s)$ unshifted reference spectrum of a 4.1 M KAcO solution (black). The contributions of electrons resulting from the ionisation from each component of the $K\alpha_{1,2}$ fluorescence doublet of $K^{2+}(1s^{-1})$ are shown in red ($^2S \rightarrow {}^2P_{1/2}$) and blue ($^2S \rightarrow {}^2P_{3/2}$). Each contribution is made of a quadruplet, like that of figure S4, with, however, a slightly worse resolution due to a spectrally broader X-fluorescence than the synchrotron source (*cf.* S3.2).

S5.2 Experimental FZRET spectrum

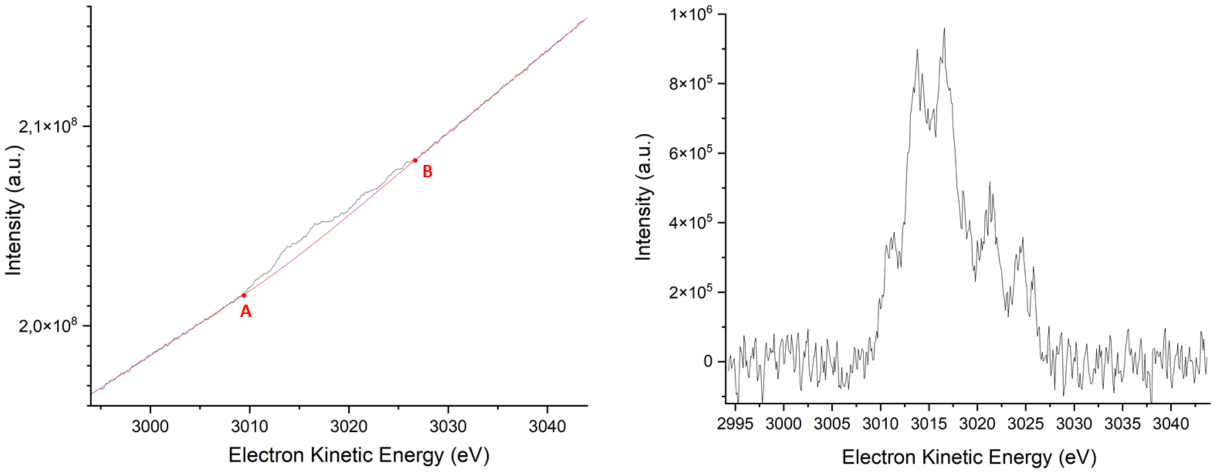

Figure S5.2.1 Photoelectron spectra (black) recorded in the spectral region expected for a FZRET signal corresponding to the $K^+(2p)/C(1s)$ ionisation by the $K\alpha_{1,2}$ photons emitted by the $K^{2+}(1s^{-1})$ ions produced by a 3800 eV synchrotron irradiation of a microjet formed from a KAcO 3 M solution. The raw spectrum is presented on the left panel together with the baseline (red) composed of two linear parts located from either side of the FZRET signal, *i.e.* below point **A** and above point **B**, and a polynomial part (between **A** and **B**), see text for explanations. The FZRET spectrum obtained after baseline correction is presented on the right panel. EKE axes are not calibrated.

The experimental FZRET spectrum of a KAcO 3 M solution recorded in the $K^+(2p)/C(1s)$ region is shown on Fig. S5.2.1. Baseline appears as linear with, however, a significant change of slope from either side of the signal (Fig. S5.2.1 left). As already explained in section 2.3.1 for such an unusual case, baseline subtraction is carried out by using the unique third polynomial connecting **A** and **B** with the relevant slopes. The baseline subtracted FZRET spectrum of a KAcO 3 M solution is presented on Fig. S5.2.1 right as well as on Fig. S5.2.2 together with the simulated unshifted FZRET reference spectrum already presented in Fig. S5.1. While both spectra appear like a quintuplet, one observes a difference in the relative intensities between the low-energy and high-energy parts of the spectra. Such a difference is actually expected as the FZRET 3 M spectrum contains the additional contribution of short-distance donor $K^{2+}(1s^{-1})$ – acceptor $K^+(2p)/C(1s)$ pairs, which produce electrons shifted to low EKE.

In order to extract the respective contributions of short- and long-distance D-A pairs, one proceeds as before (S3.4) by adjusting the relative intensity of these spectra in order to maximise their overlap, while keeping the difference signal always positive (Figure S5.2.2).

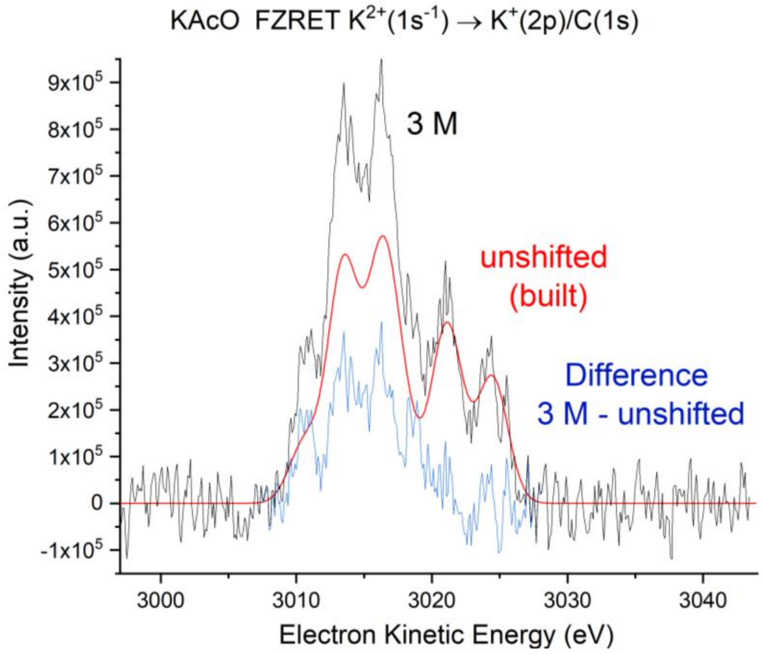

Figure S5.2.2 Baseline subtracted FZRET $K^{2+}(1s^{-1}) \rightarrow K^{+}(2p)/C(1s)$ spectrum of a microjet produced from a KAcO 3 M solution (black). Photon energy is set at 3800 eV. This spectrum can be decomposed in two contributions, that of long-distance pairs known from the simulated unshifted FZRET reference spectrum (red), and the remaining difference (blue), which corresponds to the contribution of short distance D-A pairs. See text for explanations.

S5.3 K^{+}-C pairs theoretical FZRET signal for assignment of the FZRET signal in the $K^{+}(2p)/C(1s)$ region

Similarly to S3.5, $D_d(K^{+}\text{-}C_c)$ and $D_d(K^{+}\text{-}C_m)$ are available from theoretical simulations (Section Methods and Figure S5.3).[8] They can be turned into energy distributions and used to obtain theoretical FZRET signals as already explained above (S3.5). Comparison with the difference between 3 M and unshifted FZRET signals is provided by Figure 4.

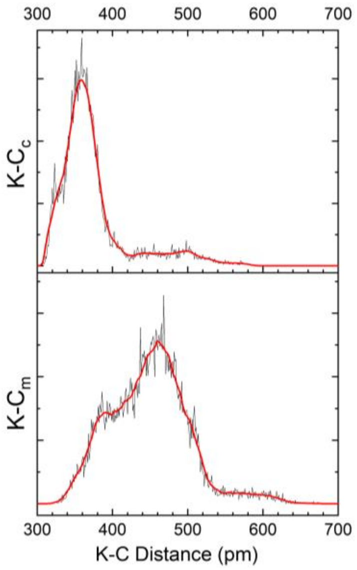

Figure S5.3 K^{+}-C_c and K^{+}-C_m distance distributions (black) for all types of ion pairs taken from ref[8]. Smoothed distributions are also shown (red).

References

1 D. Céolin, N. V. Kryzhevoi, C. Nicolas, W. Pokapanich, S. Choksakulporn, P. Songsiriritthigul, T. Saisopa, Y. Rattanachai, Y. Utsumi, J. Palaudoux, G. Ohrwall and J. P. Rueff, *Phys. Rev. Lett.*, 2017, **119**.

2 S. Thurmer, S. Malerz, F. Trinter, U. Hergenhahn, C. Lee, D. M. Neumark, G. Meijer, B. Winter and I. Wilkinson, *Chem. Sci.*, 2021, **12**, 10558.

3 D. A. Shirley, *Physical Review B*, 1972, **5**, 4709.

4 M. Lundholm, H. Siegbahn, S. Holmberg and M. Arbman, *J. Electron Spectrosc. Relat. Phenom.*, 1986, **40**, 163.

5 B. Winter, E. F. Aziz, U. Hergenhahn, M. Faubel and I. V. Hertel, *J. Chem. Phys.*, 2007, **126**.

6 C. R. Bury and G. A. Parry, *Journal of Chemical Society*, 1935, 626.

7 D. Céolin, J. P. Rueff, A. Zimin, P. Morin, V. Kimberg, S. Polyutov, H. Agren and F. Gel'mukhanov, *J. Phys. Chem. Lett.*, 2017, **8**, 2730.

8 J. Donon, S. Habka, T. Very, F. Charnay-Pouget, M. Mons, D. J. Aitken, V. Brenner and E. Gloaguen, *Chemphyschem*, 2021, **22**, 2442.

9 https://xdb.lbl.gov/Section1/Table_1-1a.htm

10 W. Pokapanich, H. Bergersen, I. L. Bradeanu, R. R. T. Marinho, A. Lindblad, S. Legendre, A. Rosso, S. Svensson, O. Bjorneholm, M. Tchaplyguine, G. Ohrwall, N. V. Kryzhevoi and L. S. Cederbaum, *J. Am. Chem. Soc.*, 2009, **131**, 7264.
